# Application of Multiparametric Intraoperative Ultrasound in Glioma Surgery

**DOI:** 10.1155/2021/6651726

**Published:** 2021-04-16

**Authors:** Ji Shi, Ye Zhang, Bing Yao, Peixin Sun, Yuanyuan Hao, Haozhe Piao, Xi Zhao

**Affiliations:** ^1^Department of Neurosurgery, Cancer Hospital of China Medical University, Shenyang 110042, China; ^2^Department of Anesthesia, Cancer Hospital of China Medical University, Shenyang 110042, China

## Abstract

Gliomas are the most invasive and fatal primary malignancy of the central nervous system that have poor prognosis, with maximal safe resection representing the gold standard for surgical treatment. To achieve gross total resection (GTR), neurosurgery relies heavily on generating continuous, real-time, intraoperative glioma descriptions based on image guidance. Given the limitations of currently available equipment, developing a real-time image-guided resection technique that provides reliable functional and anatomical information during intraoperative settings is imperative. Nowadays, the application of intraoperative ultrasound (IOUS) has been shown to improve resection rates and maximize brain function preservation. IOUS, which presents an attractive option due to its low cost, minimal operational flow interruptions, and lack of radiation exposure, is able to provide real-time localization and accurate tumor size and shape descriptions while helping distinguish residual tumors and addressing brain shift. Moreover, the application of new advancements in ultrasound technology, such as contrast-enhanced ultrasound, three-dimensional ultrasound, navigable ultrasound, ultrasound elastography, and functional ultrasound, could help to achieve GTR during glioma surgery. The current review describes current advancements in ultrasound technology and evaluates the role and limitation of IOUS in glioma surgery.

## 1. Introduction

Gliomas are among the most common highly malignant primary brain tumors, accounting for 24.7% of all primary brain tumors and 74.6% of malignant brain tumors [1, 2], with glioblastomas representing the most fatal and common type. Surgical resection followed by the Stupp protocol, which includes radiotherapy and chemotherapy with the alkylating agent temozolomide, remains the mainstay for glioma treatment [3]. The grade of glioma has been closely related to its prognosis [4], which has unfortunately remained poor despite advancements in imaging, surgical skills, and adjuvant technologies. Studies have shown that gross total resection (GTR) can positively influence quality of life, as well as progression-free survival (PFS) and overall survival (OS) [5]. Hence, the ability to achieve GTR, including precise localization and accurate tumor border description, has promoted an increase in the popularity of real-time intraoperative imaging, which is crucial for each surgical step [6, 7]. Common intraoperative diagnostic imaging techniques include intraoperative magnetic resonance (IOMR), intraoperative computed tomography (IOCT), intraoperative ultrasound (IOUS), and dyes (fluorescence). Given the problems inherent to navigation systems based on preoperative imaging, the aforementioned techniques attempt to eliminate errors produced by brain shift due to loss of cerebrospinal fluid and tumor tissue removal [8].

Over the past 10 years, the use of IOMR has verified that resection control and extent of resection promote longer survival. However, the complicated surgical arrangements, increased costs, and prolonged operative durations associated with IOMR have limited its application [9]. Recently, ultrasonography has become a widely used medical imaging modality with numerous clinical applications [10], especially in neurosurgery. Diagnostic ultrasound was first introduced into neurosurgery in the 1930s by the Dussik brothers to identify brain tumors [11]. By the 1950s, French et al. utilized the A-type ultrasound for the detection of brain tumors [12]. However, the quality of ultrasound images had somewhat restricted the widespread use of ultrasonography in neurosurgery. As such, IOUS was subsequently introduced in 1980 as a potential tool for guiding the resection of brain tumors [13]. Unfortunately, many neurosurgeons have yet to recognize this development and the ability of IOUS to address the challenges related to neurosurgery [14].

Unlike IOMR, an established imaging tool for tumor visualization and resection, IOUS has been highly regarded for its ease of use, real-time capability, portability, and affordability [7]. Moreover, IOUS adds little to the operative time. IOUS offers reliable real-time updates of the operative field (“live anatomy”) [15], which could improve intraoperative image quality in patients with glioma. Nowadays, IOUS mostly relies only on gray-scale brightness mode (B-mode) during neurosurgery [16]. Along with the rapid development of ultrasonic techniques, ultrasound is becoming a multiparametric imaging tool, which has already been used for other organs such as the liver and prostate. Several studies have confirmed that IOUS provides excellent sensitivity and specificity in predicting the diagnosis and prognosis of patients with glioma [17, 18]. Munkvold et al. found that IOUS had precise specificity but lower sensitivity compared with postoperative magnetic resonance imaging (MRI) for detecting small tumor remnants [18]. In addition, tumor volume and depth have been identified as predictors for achieving GTR. Considerable advancements in forms of new probes, contrast agents, and techniques have been made to intraoperative ultrasonography, which have made ultrasound a multiparametric imaging tool, improving surgical resection and survival rates in patients with gliomas [19]. Given the rapid development and popularization of IOUS over recent years, the current review is aimed at describing the new advancements in ultrasound technology and evaluating the role and limitations of IOUS in glioma surgery.

## 2. Ultrasonographic Characteristics of Glioma

Upon B-mode ultrasound examination, gliomas appear as single or multiple lesions with round or oval strong echoes. Considering its infiltrative growth, gliomas present with irregular margins. The classification of gliomas through B-mode ultrasound images is also of great significance. Low-grade gliomas (LGGs) are difficult to distinguish macroscopically from a normal tissue, which do not show a specific signal with 5-aminolevulinic acid [20]. LGGs are characterized by a hyperechoic and homogeneous internal echo, regular shape, clear boundary with surrounding tissue, and no obvious surrounding edema zone. These characteristics help localize subcortical lesions under a normal appearing brain surface and are important in determining the borders of LGGs [21, 22]. On the other hand, high-grade tumors (grades III–IV) have an inhomogeneous internal echo, possibly a fluid necrotic zone, irregular shape, unclear boundary, and obvious edema zone. Peritumoral edema is often resected as residual tumor tissue, resulting in unnecessary brain damage. As such, conventional B-mode ultrasound might have limited ability for providing clear descriptions of the tumor, tumor boundaries and surrounding tissue, edematous tissue, and even tumor remnants due to artifacts introduced by the resection [4, 9]. Therefore, novel IOUS techniques can be beneficial given their ability to provide real-time lesion information, distinguish between edematous and tumor tissue, and assist with the surgery. Given that LGGs have less peritumoral edema, the margins between the glioma and adjacent normal brain tissue are not covered by edematous tissue, increasing the value of ultrasound in LGGs [23]. Nonetheless, conventional B-mode ultrasound images still seem to be helpful in identifying high-grade gliomas (HGG) with obvious peritumoral edema [24].

## 3. New Developments in Intraoperative Ultrasound

### 3.1. Contrast-Enhanced Ultrasound

Contrast-enhanced ultrasound (CEUS) is a dynamic and continuous imaging modality that offers a real-time view of vascularization and flow distribution patterns of different organs and tumors, which could improve the resolution, sensitivity, and specificity of ultrasound imaging and estimation of the degree of tumor resection [25]. As a functional imaging equipment, CEUS quantitatively evaluates solid tumor perfusion using a mathematical pattern that analyzes raw linear ultrasound information [26]. Contrast agents have been widely used to quantify the flow characteristics through an organ or tumor given their ability to highlight the microcirculation [27]. The contrast agent used during CEUS contains microbubbles, which have been demonstrated to provide the highest contrast during ultrasound scanning [27, 28]. These microbubbles are encapsulated in a layer of protein or polymers, which are comprised of air or inert gas, such as perfluorocarbon or nitrogen [29]. The microbubbles are too small to be filtered by the lungs and can be transported into the smallest capillaries, thereby allowing visualization of the arterial system after venous injection [29]. This contrast enhancement technique facilitates real-time evaluation of the tumor, vascularity, and tissue perfusion. Moreover, CEUS helps locate the tumor and aids clinicians in accurately and immediately determining the relationship between internal blood vessels and peripheral blood vessels [4, 30].

Although CEUS is a valuable diagnostic tool that has already been widely utilized for the liver, kidney, and other organs [31], its application in the field of glioma surgery has been limited (Table 1). In 1993, Bogdahn et al. had been the first to utilize CEUS to detect brain tumor neovascularization during brain surgery [32]. CEUS can provide important real-time imaging patterns during glioma surgery. Indeed, Prada et al. found that different brain tumors can be characterized by different contrast-enhanced patterns [29]. Accordingly, LGGs show a mild, dotted contrast-enhanced image with diffuse appearance and slower, delayed arterial and venous phase, whereas HGGs have a high contrast-enhanced image with a more nodular, nonhomogeneous appearance and fast perfusion patterns [29]. Given the different blood supply types between tumors and normal brain tissues, CEUS can better locate and demarcate glioma borders compared with conventional ultrasound while also identifying glioma blood vessels [33]. This is of great significance when determining residual tumors and the peritumoral edema zone after tumor resection. Most of the tumor boundaries displayed using CEUS are larger than those displayed using conventional ultrasound. Therefore, CEUS has more clinical significance when imaging HGGs. Contrast agents have varying residence times in benign and malignant brain tumors. As such, distinguishing benign and malignant tumors according to the contrast agent development time-intensity curve is definitely valuable [4]. Although CEUS can help identify residual tumors during glioma surgery according to the changes in local enhancement [34], the application of contrast-enhanced IOUS has been restricted to estimating the degree of tumor removal among patients with glioma or recurrent glioma after radiotherapy given that recurrent tumors, radioactive necrotic tissues, and peripheral edema tissues manifest similar medium-strong echoes [25]. Recently, Cheng et al. found that certain CEUS parameters, such as time to start (TTS) and time to peak (TTP), can be used to determine tumor grade [4]. Accordingly, the TTS and TTP of LGGs resembled those of peritumoral edema and normal brain tissue, with LGGs have greater enhancement. On the other hand, the TTS and TTP of HGGs were earlier than those of edematous and normal brain tissue [4]. This indicates that glioma boundaries are clearer than peritumoral edematous brain tissue and surrounding normal brain tissue, thereby suggesting that CEUS can be used to improve the identification of glioma, peritumoral edema, and normal brain tissue [35]. Moreover, the absolute peak intensity had a positive correlation with microvessel density (MVD), which is a quantitative criterion that objectively reflects the proliferation and angiogenesis of tumor cells, angiogenesis, and degree of malignancy [4, 36]. These quantitative parameters of CEUS may indirectly indicate the hemodynamic features and MVD of gliomas, which help reliably and noninvasively estimate glioma angiogenesis, evaluate pathological grade, and offer meaningful information that could guide surgical strategies and postoperative treatment [36, 37].

Previous studies have found that gliomas grow rapidly and are rich in peripheral blood vessels but have immature differentiation, incomplete collateral circulation, and high vascular permeability, all of which led to the better identification of edematous tissue [38, 39]. Peritumoral edema is an important cause of glioma recurrence. Intraoperative CEUS can effectively distinguish between high-intensity tumor tissue and equal-intensity peritumoral edema, provide the basis for tumor resection, and protect normal brain tissue to the maximum possible extent [25]. Moreover, CEUS allows clinicians to estimate the function and anatomy of the vascular tree.

CEUS permits real-time angiosonography, which offers useful information on the parenchyma, as well as tumor vascularization and perfusion. CEUS can also help in glioma detection and surgical planning, which also allows tumor grade characterization and residual identification [16]. Therefore, the value of CEUS lies in its ability to estimate tumor location, evaluate boundaries, distinguish benign and malignant lesions, achieve maximal tumor resection, and improve disease-free survival rates in patients with gliomas [25, 36]. Thus, the utilization of intraoperative CEUS in neurosurgery can only be expected to increase.

### 3.2. Three-Dimensional Ultrasound

Three-dimensional ultrasound (3DUS) is based on two-dimensional ultrasound (2DUS) imaging with the addition of the coronal section. Intraoperative 3DUS is a portable imaging technique that provides real-time imaging, which can be integrated into existing theater infrastructure without the need for its own dedicated suite [7]. Recently, 3DUS imaging has already been used for cardiac, obstetric, and gynecological applications [42]. As neurosurgery is the sole field where 3DUS has such a large application, it has currently been used widely in neurosurgery, particularly for intraparenchymal brain tumors (Table 2), cavernomas, skull base tumors, medulla lesions, arteriovenous malformations, and endoscopy guidance [14]. 3DUS can complete a series of functions, including stereoimaging, image segmentation, image rotation, and high-plane image analysis [43]. Given that reconstructed images are clear and intuitive, they can be used to measure the size and volume of gliomas, as well as clearly display the 3D shape and spatial location of the lesion. This technique could provide accurate visualization of blood vessels, the ventricular system, lesions, and immediate complications, such as hematomas [7]. 3DUS can not only make up for the spatial imaging deficiency of two-dimensional ultrasound but also reduce artifacts produced after tumor resection [44]. Another benefit of 3DUS is its ability to provide new real-time images, which enable neurosurgeons to operate with updated and accurate volumes during surgery. Surgeons can obtain new image volumes relatively quickly, which would then allow the surgery to proceed according to the new precise volume [45]. Moreover, during insular LGG surgery, navigable 3DUS can help prevent lenticulostriate artery (LSA) injury by visualizing the location and shape of the LSAs [46].

Camp et al. found that mean pixel brightness (MPB) objectively quantifies echogenicity and standard deviation (SD) heterogeneity [7]. The highest MPB values were observed at the solid part of HGGs, whereas the necrotic core and infiltrating margins of the tumors were found to have low MPB values. The infiltrating margins of the HGGs were found to have higher SD values [7], which were also raised in areas of varying cellularity within the same tumors and within solid HGGs (World Health Organization grade IV) with impending necrosis [7]. MPB and SD are objective parameters mirroring the sensitivity of 3DUS in detecting the presence and extent of gliomas. Moreover, they could be used to indirectly suggest heterogeneity, cellularity, and invasiveness, offering information regarding the nature of the tumor, as well as reflect the sensitivity of IOUS in detecting the presence of residual tumors [47]. Furthermore, surgeons could sample and analyze SD values at the tumor margins in real-time, which offer information for designing a possible surgical plane [7]. The aforementioned findings highlight the application of 3DUS and its utility as a potential therapeutic technique during glioma resection. Indeed, observing the dynamic surgical changes in real-time is convenient. Moreover, given that tumor always pushes the tract away, LGGs in the eloquent area depicted by 3DUS can be resected without risking permanent neurological deficits [43]. For HGGs located at eloquent areas, resection can be done with very low morbidity and prominent improvement of function [48].

At present, 3DUS combined with navigation systems or CEUS has been widely used in brain surgery, which sheds meaningful light on the maximal safe resection during glioma surgery. Nowadays, neuronavigation systems have been used ubiquitously for clinical biopsies, which are mainly based on preoperative images. However, patient registration procedures and brain shift have promoted navigational inaccuracies. Through 3DUS, all such sources of error can be largely eliminated given that 3DUS provides volume measurements immediately before biopsy collection [49]. Performing 3DUS angiography for gliomas located at vascular regions can help avoid vessel injuries caused by the biopsy forceps. Moreover, generating 3DUS volume measurements after biopsy sampling is important for modifying and locating the best biopsy site [49]. Unfortunately, the success rate of 3DUS-based stereotaxis has yet to be compared to those of MR-guided biopsies.

A previous study has shown that navigable 3DUS-guided biopsy is as good as navigable MR in defining the tumor boundaries [50]. Notably, several neurosurgery centers have imported intraoperative 3DUS, with integrated image guidance based on multimodal preoperative imaging, to achieve GTR of brain tumors [7]. Bø et al. found that 3DUS-guided LGG resections under general anesthesia were safe and preserved health-related quality of life in most patients [51]. Meanwhile, Sæther et al. proved that patients with glioma displayed improved survival around the same period that IOUS and neuronavigation had been introduced [44]. Moreover, clinical trials have been designed to verify the effectiveness of 3D reconstruction and CEUS in the resection of brain tumors using ultrasound contrast agents. Accordingly, their results indicated that the combination of 3DUS and CEUS provided more advantages during glioma surgery and that the combination pattern seems to be a popular research topic worldwide [9]. Studies have shown that 3D-CEUS is a reliable intraoperative imaging modality that helps enhance imaging quality [9]. Most HGGs exhibited high contrast uptake, subsequently increasing imaging quality in more than 50% of the cases. As such, GTR and incomplete glioblastoma resection were sufficiently highlighted by 3D-CEUS intraoperatively. Moreover, the use of ultrasound contrast agents for 3D-CEUS seemed to have provided better imaging patterns, especially for resection control in glioma surgery. The aforementioned results underscore the value of 3DUS in glioma surgery. At present, image quality has still remained a constraint for the future use of 3DUS in glioma surgery. Despite recent improvements in image quality, an obvious potential for further refinement does exist [14]. Taken together, 3DUS can be considered a cost-effective, reliable, portable, and real-time intraoperative imaging modality that minimizes injury, improves surgeon efficiency and confidence when conducting glioma surgery, and guides the selection of surgical treatment options for glioma [7]. Therefore, 3DUS can be used not only as a diagnostic tool but also to guide surgical strategies. To obtain the optimal benefit from 3DUS, neurosurgeons should master more principles for utilizing IOUS in glioma surgery.

### 3.3. Navigable Ultrasound

Conventional navigation systems have been widely used to facilitate accurate planning and execution of tailored craniotomies, which are mainly based on preoperatively acquired imaging data [60]. Moreover, this technique helps with tumor localization and planning of the optimal entry path to the lesion. However, conventional navigation systems cannot mirror brain shift that occurs during glioma surgery, making preoperatively acquired images inaccurate and, therefore, no longer reliable for further navigation.

Navigable ultrasound (NUS) is a new technology that allows for localization and navigation of tumors by tracking two-dimensional (2D) or 3D ultrasound images [55]. Moreover, this technique is an innovative intraoperative imaging adjunct that allows for quick real-time updates to facilitate tumor resection (Table 3). Moiraghi et al. proved that real-time NUS promoted better extent of resection (EOR) and neurological outcomes during resection of noneloquent HGGs compared with conventional neuronavigation (NN). Moreover, NUS has verified usefulness in detecting residual tumors with a volume of >1 cm^3^ [61]. Another study showed that the integration of 3DUS with neuronavigation technology resulted in an efficient and inexpensive imaging modality for intraoperative neurosurgery imaging that can be used in conjunction with preoperative MR images [43]. Meanwhile, Moiyadi and Shetty demonstrated that 3DUS can be effectively used as a stand-alone navigation system during the resection of gliomas without the need for preoperative MRI [55]. NUS has mainly been based on 3DUS. To produce 3D images, a precalibrated 2D tracked phased array probe can be used. The 3D volume is reconstructed by acquiring 200–300 images while tilting the probe, which can then be utilized for the purposes of navigation [62]. Recently, the innovation of 3DUS probes has allowed the acquisition of 3D datasets without the need for volume reconstruction using 2D slices [63]. The integration of a 3D probe into the neuronavigation system has certain advantages over a 2D probe.

Navigable 3DUS can overcome issues related to orientation and permits precise intraoperative imaging given its short acquisition times when updating 3D volumes. Lindseth et al. thoroughly investigated the actual clinical precision of the IOUS-based neuronavigation system (SonoWand) [49]. Accordingly, the experimental accuracy was estimated to be 1.40 ± 0.45 mm, whereas the accuracy based on clinical data from tissue slices was found to be below 2 mm. Moreover, Solheim et al. utilized the SonoWand 3DUS navigation system, which provides patient-related orthogonal sections and tracker-related reconstruction images, in glioma surgery [64]. Their results showed that the removal rate of HGGs with a 3D navigation system could be as high as 92%. Given that optimal resection of brain tumors located at eloquent areas requires some combination between intraoperative imaging and functional monitoring during surgery, combining awake surgery with NUS may be feasible and beneficial [54]. After comparing 3D-NUS-guided glioma surgeries and conventional navigation, Šteňo et al. revealed that the former achieved a greater extent of awake resections of eloquent LGGs than the latter, with the use of 3D-NUS having no influence on the number of new permanent deficits [53]. Coburger et al. demonstrated that NUS can be a safe and accurate imaging technique for guiding resection control of glioblastomas. Compared with conventional IOUS, NUS exhibited a prominently better residual tumor detection rate [65].

Moreover, Allouch et al. demonstrated that NUS-guided biopsies achieved a similar percentage of conclusive diagnoses compared with stereotactic biopsies [66]. In the aforementioned series, which has been the largest research on NUS-guided biopsies, Allouch et al. utilized 2DUS coupled with a biopsy guide and superimposed the trajectory on the sonograms for guidance, achieving a diagnostic yield of 92% [66]. Patil et al. obtained a similar yield of 92.8% in their study of 125 NUS-guided biopsies of supratentorial tumors [67]. The direct use of NUS can have more advantages and benefits, including repeated, high-quality intraoperative updates that can be helpful and useful for guiding glioma biopsy and resection, as well as brain shift control [67]. Notably, just as preoperative MR images can be navigated, ultrasound images can also be tracked and navigated through NUS, which is essentially a “tracked” ultrasound. Direct NUS could be used as a stand-alone system, which decreases the need for preoperative MRI [55] and saves more time and money. It can also be especially useful in emergency cases wherein MRI could hardly be used. Direct NUS helps avoid registration inaccuracies inherent to image-to-patient registration algorithms given that image acquisition and display are done in the same reference frame. Given the ease and convenience of obtaining NUS images, they can be repeated as many times as required without significantly prolonging the overall duration of surgery. Direct NUS is inexpensive and does not interfere with the surgical workflow, which can be particularly convenient in awake surgeries. Compared to other real-time intraoperative imaging modalities, direct NUS offers an equally effective alternative to frame-based or frameless stereotactic biopsy, with the added benefit of real-time monitoring of postprocedure intratumoral bleeding [67]. However, direct NUS still has its shortcomings. The first challenge neurosurgeons face concerns the interpretation of ultrasound images, especially for less experienced surgeons. Large 3D volumes can be reconstructed by including surrounding brain and known anatomical landmarks (apart from the tumor area), which considerably helps surgeons determine the orientation. As mentioned earlier, preoperative MR images can be applied during the early phase of the surgeon's learning curve. 3D-NUS is a novel intraoperative imaging strategy for stand-alone navigation during glioma surgery [55]. Despite its suboptimal image quality, meticulous technique can minimize suboptimal image quality in several cases. 3D-NUS offers fast, precise, and real-time updates and facilitates optimal resection without significant financial or logistical challenges. Experience with NUS image acquisition and interpretation is essential for increasing image quality and extracting the maximum benefit from this potentially useful adjunct.

### 3.4. Ultrasound Elastography

Ultrasonography had been first introduced into clinical practice in the 1970s [10]. Since then, new ultrasound techniques have been developed, such as Doppler imaging, which offers new information for diagnosis and provides real-time control of tumor resection [69]. Elastography, which is the science of creating noninvasive images of the mechanical characteristics of tissues, has been rapidly evolving recently (Table 4). The concept of “elastic imaging” was first mentioned in 1990 [11, 13, 70]. By applying internal or external excitation to the tissues, which produced different responses, ultrasonic waves were able to obtain corresponding ultrasonic signals by monitoring the movement process of these tissues. Subsequently, ultrasound elastography (UE) was used to map tissue stiffness and reproduce the palpation performed by surgeons. During neurosurgery, real-time brain elasticity imaging could offer new intraoperative images for tumor localization and boundary assessment [71]. Strain elastography estimates tissue macrostructure, while conventional ultrasound characterizes tissue elasticity at a different level [62]. Elastic tissue parameters were obtained using an elastic imaging algorithm. Recently, UE has been widely used for evaluating traumatic brain injuries, ischemic stroke, and hypoxic ischemic encephalopathy, especially in intraoperative brain tumors [72, 73]. Macé et al. first measured the elastic modulus of rats' brain using elastic imaging in 2011 [71]. Considering the size of a mouse brain, estimating elasticity values of different areas is difficult. Lay et al. used high-frequency UE based on shear-wave imaging to map cortical stiffness in mice of different ages [74], although no statistical difference was found. In addition, changes in the elastic modulus caused by pathological changes can be used to assist in intraoperative localization and diagnosis. Moreover, Bal et al. revealed that UE combined with conventional B-mode ultrasound may be a valuable adjunct to distinguish tumors from normal brain tissue [62]. Sebag et al. also supported its use to better differentiate normal tissues from brain tumors [75]. Notably, ultrasound-based elastography techniques comprise transient elastography, acoustic radiation force impulse elastography, shear-wave elastography (SWE), and strain or real-time elastography [17]. Recently, SWE has enabled the examination of living tissue stiffness, which has been considered more quantitative, objective, and reproducible [76]. SWE uses an acoustic radiation force pulse sequence to create shear waves that propagate perpendicular to the ultrasound beam, resulting in transient displacements [77]. The distribution of shear-wave velocities at each pixel is directly related to the shear modulus, an absolute measure of the tissue's elastic values [76]. Shear-wave images are automatically coregistered with standard B-mode images, producing quantitative color elastograms with anatomical specificity. Moreover, shear waves propagate faster through stiffer contracted tissue [76]. Therefore, in 2015, Chauvet et al. used SWE to evaluate gliomas. Interestingly, Young's modulus (the physical parameter corresponding to stiffness) measured using SWE provided new insights into the differentiation of brain tumors, with the LGG and HGG subgroups showing a stiffness of33.1 ± 5.9and23.7 ± 4.9 kPa, respectively [78]. Normal brain tissue has been described by a reproducible mean stiffness of 7.3 ± 2.1 kPa. Notably, the stiffness of LGG differed from that of HGG (*p* = 0.01), while normal brain tissue stiffness significantly differed from that of LGGs (*p* < 0.01) [78]. Previous studies have shown obvious differences in elasticity values among common brain tumors. Thus, neurosurgeons could use intraoperative SWE, which offers more innovative information, for establishing a diagnosis and guiding tumor resection [78].

Recent studies have verified that all stiffness images have the same scale ranging from 1 kPa (dark blue) to 90 kPa (dark red). Colors like red, yellow, and cyan can indicate benign tumors, while only dark blue can indicate malignant tumors [79, 80]. Previous studies utilizing a real-time elastic imaging technique during surgery found that the color signal of normal brain tissues was mainly green and uniformly distributed, whereas LGGs showed a clear boundary with mostly green signals [79, 80]. On the other hand, majority of the HGGs showed a blue signal with red and green signals inside, while edematous areas showed a red signal around the tumor. Briefly, previous research found that UE offers substantial advantages over conventional ultrasound in contrast resolution [80, 81]. Although conventional ultrasound offered the highest sensitivity, strain images and strain ratio offered higher specificity [80]. Recently, Cepeda et al. used UE to determine the elastographic patterns across different types of brain tumors and determined differences between their peritumoral regions [17]. Using receiver operating characteristic curves, they obtained optimal cutoff points of 92.22 (whole tumors) and 109.6 (peritumoral regions), which suggested that tumors having values below these cutoff points had a high probability of being a glioma [17]. As such, UE can be a promising modality for better defining tumor boundaries, parenchymal infiltration, tumor consistency, and tumor stiffness while also allowing the differentiation of high- and low-grade lesions [16]. Therefore, UE can provide more information within the tumor, help surgeons determine the nature of the tumor, and improve removal rates.

### 3.5. Functional Ultrasound

Infiltrative growth, one of the main features of gliomas, often interferes with the eloquent area. Thus, functional mapping of brain activity is very important for the removal of gliomas, reduction of postoperative complications, and improvement of patients' quality of life [82]. However, functional neuroimaging cannot provide images of neural activity within the deep portions of the brain, while 2D ultrasound lacks the sensitivity for discovering blood flow in small vessels where majority of hemodynamic responses occur [42].

Therefore, combining neurofunctional imaging with ultrasound can be an optimal option [83]. FUS, a new mobile neuroimaging modality with unprecedented spatiotemporal resolution [84–86], permits brain activity imaging by monitoring transient changes in cerebral blood volume dynamics that mirror the changes in metabolic activity of activated neurons through neurovascular coupling (Table 5). Notably, Mace et al. demonstrated that FUS allowed for precise localization of the functional area of the cerebral cortex, which can help facilitate maximum tumor resection and preserve the functional area, thereby reducing postoperative complications and improving patients' quality of life [83]. FUS uses plane-wave illumination at an ultrafast frame rate (1 kHz), allowing the measurement of transient changes in cerebral blood volume with a high spatiotemporal resolution (250 *μ*m, 1 ms) [70, 84]. FUS can be utilized to confirm brain activity areas based on increased cerebral blood volume due to neurovascular coupling. Imbault et al. demonstrated that FUS can identify, map, and distinguish brain activation areas in two dimensions during task-evoked cortical responses within the depth of a sulcus in both awake and anesthetized patients [83, 84].

Soloukey et al. found that the relevance of FUS for awake brain surgery lies in its ability to capture both task-evoked functional cortical responses and differences in vascular characteristics between tumor and healthy tissues [86]. Unfortunately, current neurosurgical practice still predominantly leans toward inherently limited preoperative imaging tools for guiding tumor resection. After entering the scene, FUS has been a promising alternative that provides both anatomical and physiological data [86]. Although FUS is currently restricted to 2D, high-resolution 3DUS images of the brain vasculature can be expected in the very near future, which will definitely provide greater advantage. Moreover, Demené et al. recently extended the capabilities of FUS to whole-brain four-dimensional vascular neuroimaging in rodents using a tomographic approach, which will be of great value in the study of human brain hemodynamics [87, 88]. To allow for blood flow velocity imaging of the entire rodent brain, Tang et al. recently developed high-speed, contrast-free, quantitative ultrasound velocimetry (vUS) based on the normalized first-order temporal autocorrelation function of the ultrasound field signal [89, 90]. vUS can quantify blood flow velocity in both the transverse and axial directions, which had been validated using numerical simulation, phantom tests, and in vivo measurements [89, 90]. The functional imaging ability of vUS can be verified by monitoring the blood flow velocity changes during whisker stimulation in awake mice [89]. Unlike existing FUS imaging tools, vUS displayed quantitative accuracy in assessing both axial and transverse flow speeds and resistance to acoustic attenuation and high-frequency noise [89]. Thus, FUS and related technologies seem to promote substantial changes in glioma surgery.

## 4. Application of Intraoperative Ultrasound in Glioma Surgery

The main objective of brain tumor surgery is to remove as much tumor tissue as possible and reduce recurrence rates. Intraoperative navigation has become the standard for preresection initial localization and evaluation of tumor boundaries in many hospitals. Multiparametric IOUS is attractive due to its abovementioned advantages [6] and has been mainly used in (1) intraoperative navigation, (2) evaluation of resection range, and (3) monitoring of brain shift.

### 4.1. Intraoperative Navigation

Neuronavigation has improved patient outcomes given its enhanced accuracy and safety during brain surgery [91]. Considering that brain shift is not accounted for by preoperative imaging, real-time intraoperative imaging remains important. IOUS has the ability to accurately localize the brain tumor, which helps reduce invasiveness and therefore potential surgical morbidity, and permits minimally invasive procedures, which is important in lesion localization, approach planning, and biopsy of intracranial lesions [52, 67]. Unlike other modalities, IOUS is a portable system offering real-time imaging for intraoperative navigation, which has been achieved using both 2D and 3D ultrasound [62]. Furthermore, navigable 3DUS addresses the issue of orientation and allows accurate intraoperative imaging. The ability of real-time IOUS to update preoperative images allows it to guide surgery. Given the ease of obtaining new information for navigation, real-time updated image volumes can be used to guide the entire surgical course. The increased precision provided by 3DUS systems, based on nearly real-time images, permits accurate and safe resections, which is especially useful and meaningful in surgeries performed through quite narrow transcortical corridors [45]. Meanwhile, Patil et al. proved that despite the longer operative time and higher related complication rates, NUS may be beneficial and appropriate for biopsies of deep-seated gliomas [67]. With the technological advancements in ultrasound imaging (such as CEUS, 3DUS, NUS, and UE), real-time multiparametric intraoperative imaging to guide surgery has become widely available and relevant.

### 4.2. Evaluation of the EOR

Assessing the extent of excision helps improve survival rates of patients with gliomas given that even experienced neurosurgeons can make mistakes in its assessment [92]. Therefore, evaluating the EOR during surgery can be used as a key auxiliary tool. After using B-mode IOUS in 25 pediatric patients with brain tumors, El Beltagy et al. [93] revealed that B-mode IOUS images during surgery allowed for improved discernibility of tumor and postresection residual tumor margins from normal brain tissues. Moreover, B-mode IOUS provided better distinction between brain tumors and normal brain tissues in all cases, which aided lesion resection. Policicchio et al. also proved that B-mode IOUS had higher sensitivity for detecting most brain tumors [52]. As such, conventional B-mode IOUS seems to be a safe and accurate modality for planning trajectories into intraparenchymal lesions (including minimally mini-invasive approaches) while allowing for precise assessment of the EOR in more than 80% of the cases. Thus, B-mode IOUS is a versatile and practical technique that could improve safety and may be recommended for routine glioma surgery [52]. Although B-mode IOUS can provide real-time localization updates before resection of gliomas, its ability to reliably distinguish residual tumors is limited by a number of factors. Peritumoral edema produces echoes similar to the surrounding normal brain tissue, leading to difficulties in determining the actual boundary of the tumor. Moreover, bleeding at the margin of the tumor cavity interferes with the surgeon's decisions. Thus, comparing the scope of the tumor before surgery and the size of the tumor cavity after being filled with normal saline under a microscope is necessary to determine whether the tumor is residual. Woydt et al. [24] and Chacko et al. [94] compared 2DUS imaging information after resection with tumor pathologies using probes at several points within the resection boundaries. Accordingly, their findings revealed that 2DUS has high specificity for detecting residual tumor and could therefore improve GTR rates. Moreover, 2D ultrasonography was able to reliably detect residual tumors larger than 1 cm but not smaller residues. The investigation of navigable 3D-IOUS revealed a limitation similar to that for 2D ultrasound.

Meanwhile, resection is of great importance for the treatment of LGGs given that it hinders malignant transformation and improves patient survival. However, under conventional conditions, distinguishing gliomas from normal brain tissue has always been challenging. Unsgård and Lindseth revealed that the improved tumor control obtained during 3DUS-guided LGG resection was not achieved at the expense of patient quality of life [45]. Rygh et al. demonstrated that before resection, ultrasound was able to describe the tumor boundary with high specificity and sensitivity (both 95%) [50]. However, 3DUS had been found to have acceptable sensitivity (87%) but poor specificity (42%) during resection and poor sensitivity (26%) but acceptable specificity (88%) after resection [50]. Meanwhile, Liang et al. reported that B-mode IOUS-guided glioma resection had an accuracy of 96.2% [95]. False positivity during tumor resection can be caused by edema and boundary artifacts. Given that CEUS provides better intraoperative detection of tumors compared to 2D ultrasound, it can be used for preoperative exploration and evaluation of residual tumors. Moreover, CEUS provides information on the distribution of blood vessels, which helps determine the EOR and preoperatively evaluate tumors and edema [96]. Performing CEUS again after tumor resection revealed a mass or band of strong echo foci surrounding the cavity of residual tumors, if indeed present. However, when no residual tumors were present, a clear, smooth edge around the tumor cavity without an abnormally strong echo was noted [40]. The ability of CEUS to differentiate gliomas and artifacts from normal tissue on B-mode is based on its ability to determine the degree of vascularization and not the echogenicity of the tissues. Thus, CEUS plays a significant role in the maximal safe resection of glioblastomas [40]. Recently, Della Pepa et al. used both 5-aminolevulinic acid and CEUS, which they called “Enhancing Vision,” to improve radicality in glioma surgery. Accordingly, they confirmed that such a combination promotes better residual tumor identification compared with conventional microsurgical procedures [97]. Moreover, 5-aminolevulinic acid- and CEUS-guided surgeries had a positive impact on the EOR, OS, and PFS [97]. Unsgaard et al. demonstrated that 3DUS had higher sensitivity in detecting tumor remnants than with bare eyes, thereby helping achieve GTR [59]. Thus, multiparametric IOUS has been regarded as a useful and promising imaging modality for defining the margin between gliomas and normal brain tissues before resection, assessing tumor residuals after resection, and helping surgeons determine the shortest and safest access to the glioma during surgery [98].

### 4.3. Monitoring of Brain Shift

During surgery, the morphology of the brain may deviate from that delineated on the preoperative images once the dura is opened. This phenomenon, which is called “brain shift,” may lead to inaccuracies and still remains a major concern in neurosurgery [99]. During traditional surgery, surgeons mainly plan incisions based on preoperative imaging data and rich clinical experience. For subcortical lesions, however, planning is mainly based on whether the sulcus becomes shallow, the texture, and the color of the local brain tissue [100]. Occasionally, brain puncture may be needed to confirm tumors with cystic lesions. However, these indirect signs do not provide an intuitive understanding of the spatial location of the tumor. Nonetheless, the recent development of neuronavigation technology has allowed the linking of preoperative imaging data with the accurate location of the lesion during surgery using a computer, which can locate the lesion in real time and subsequently improve surgical accuracy [101]. However, the application of neuronavigation during surgical resection has been limited by the loss of cerebrospinal fluid and displacement of brain tissue. As a promising approach, multiparametric IOUS using advanced registration algorithms can update the data in real time, which helps address the problem of brain shift [102]. The primary advantage of real-time 2DUS is not only the unlimited repetition as necessary but also the ability to overcome the limit of anatomic distortion due to brain shift and tumor debulking, which also avoids the cost and duration of other intraoperative techniques [58, 103]. Prada et al. found that brain shift distortion may be corrected by the fusion of images between 2DUS and a neuronavigator, which is reliable, accurate, and easy to use, allowing a continuous real-time feedback without interrupting surgery [6, 104, 105]. Furthermore, Unsgård et al. revealed that 3DUS was accurate (<2 mm off) in lesions with sharp margins, whereas conventional navigation based on preoperative MRI was off by 2–10 mm from the actual tumor position [43]. Riva and Hennersperger reported that 3DUS can help improve registration precision by 29.2% and 33.3% after dural opening and 5.2% and 0.4% after resection through rigid and elastic registrations, respectively [102]. Therefore, 3DUS can improve the detection of brain shift, which helps to significantly increase the precision of the navigation. Moreover, Sæther et al. demonstrated that the combined application of IOUS and navigation could improve survival from 9.6 months to 11.9 months (HR = 0.7; *p* = 0.034) [44] and correct the problem of brain shift navigation. This is especially suitable for the surgical excision of small deep lesions and adjacent functional areas.

## 5. Limitation of Intraoperative Ultrasound

Despite that IOUS is a versatile, cost-effective, and efficient imaging modality [62], its utilization has still remained limited. This may be attributed to the learning curve for obtaining ultrasound images among surgeons, poor resolution, lack of uniform intracranial ultrasonography guidelines, or the difficulty in obtaining the images for smaller or deeper tumors [97]. For better surgical practice, it is pivotal for neurosurgeons to undergo abundant ultrasound trainings and learn related processes in order to obtain optimal benefit [13, 43]. Additionally, artifacts produced during surgery are often indistinguishable from residual tumors, with brain contusions or blood clots possibly also causing strong echoes at the bottom of the residual cavity after tumor resection. These become more important during glioma resection due to their shadowing effect on the tumor cavity [106]. Glioma resection may also create ultrasound image noise when the resection cavity is filled with isotonic saline water. The limitations of ultrasound in delineating residual tumors are mainly related to two issues: image quality and anatomical orientation [55]. Although advancements in ultrasound technologies have significantly improved resolution, a certain amount of training and experience, which can be easily obtained, is still needed before optimal use can be expected. Furthermore, studies have shown that B-mode IOUS resolution and quality needed to identify tumor remnants at the resection cavity boundaries decreased significantly to the point of unreliability after resection, mainly due to surgical artifacts, although several methods have been developed to reduce the artifacts [21]. Selbekk et al. reported that artifacts are mainly caused by differences in the attenuation of the resection cavity fluid and the surrounding brain [107]. The effects of artifacts can be reduced by inserting the ultrasound probe with small footprints into the resected cavity and observing suspected areas using a close-up view. One study verified the potential of a novel acoustic coupling fluid to decrease surgically induced ultrasound artifacts to a minimum during IOUS-guided brain tumor surgery [108]. Another larger problem, however, has been the lack of anatomical orientation.

The absence of known anatomical landmarks makes it hard to determine orientation, especially when attempting to observe small areas of residual tumor. Although real-time ultrasonic scanning could technically overcome this problem, inaccuracies may also emerge if the tip of the surgical tool is inaccurately placed in the insonation plane. Overall, interpreting images during the process of tumor resection remains difficult [62]. Miniaturization of probes may improve the resolution of images. Nonetheless, with the advancements in image quality and anatomical orientation, the aforementioned hurdles can soon be overcome.

## 6. Conclusion

GTR of gliomas remains the most important factor that determines patient survival and quality of life. Given the invasive nature of gliomas, accurately determining their margins through color and texture observations via the naked eye or microscopy has remained difficult. The development of neuronavigation technology has brought significant advantages in the surgical resection of brain tumors, whereas brain shift after craniotomy remains limited in its use. Owing to its relatively high sensitivity and specificity, multiparametric IOUS may overcome these limitations to some extent and become an important tool for neurosurgery [6]. Moreover, advancements in ultrasound techniques may improve the resolution and quality of ultrasound images, reduce artifacts, and improve the histological diagnosis of brain tumors. Currently, advancements in ultrasound image processing, along with continuous hardware improvements, are urgently needed to extract the full potential of IOUS [16]. In conclusion, IOUS can be utilized during routine intracranial glioma resection to improve tumor excision, promote more radical resection, and finally enhance patient survival and quality of life.

## Figures and Tables

**Table 1:** Summary of the applications of contrast-enhanced ultrasound (CEUS) for glioma.

Year	First author and country	Patient no.	Grade	Study endpoints	Results
2019	Wang et al. (China) [36]	49	HGG 23 LGG 26	To analyze the relationship between quantitative CEUS parameters and microvessel density (MVD) in different grades of gliomas	CEUS provides dynamic and continuous real-time imaging and quantitative data analysis of different grades of gliomas; the quantitative CEUS parameters were closely related to MVD and were helpful in understanding glioma grade and optimizing surgical strategy
2016	Prada et al. (Italy) [40]	10	HGG 10	To assess the capability of CEUS to identify residual tumor mass during glioma surgery and to increase the extent of resection (EOR)	CEUS is extremely specific in the identification of residual tumor. The ability of CEUS to distinguish between tumor and artifacts or normal brain on B-mode is based on its ability to determine the vascularization degree. Therefore, CEUS can play a decisive role in the process of maximizing GBM resection
2016	Lekht et al. (USA) [41]	5	HGG 1 LGG 1 Others 3	To provide further clinical data on the versatile application of CEUS through a technical note and illustrative case series	CEUS provides safe, real-time, and dynamic contrast-based imaging that can potentially be used for routine neurooncological surgery and image-guided biopsy. CEUS eliminates the effect of anatomical distortions associated with standard neuronavigation and provides quantitative perfusion data in real time, which may hold major implications for intraoperative diagnosis, tissue differentiation, and quantification of EOR
2016	Cheng et al. (China) [4]	88	HGG 50 LGG 38	To investigate the value of CEUS for evaluating the grade of glioma and the correlation between MVD and vascular endothelial growth factor (VEGF)	CEUS could help determine the boundary of peritumoral brain edema of glioma. CEUS parameters in cerebral gliomas could indirectly reflect MVD and VEGF
2015	Yu et al. (China) [25]	120	HGG 76 LGG 44	To evaluate the diagnostic significance of CEUS in assessing the resection degree of brain glioma using transmission electron microscopic (TEM) examination	CEUS had high sensitivity and specificity for evaluating the extent of tumor excision. Residual tumor rates detected using ultrasound contrast and TEM examination, respectively, had medium consistency. The application of intraoperative contrast-enhanced ultrasound can improve the resection rate of brain glioma
2014	Prada et al. (Italy) [33]	71	HGG 37 LGG 16 Others 18	To evaluate and describe different brain pathologies using CEUS compared to preliminary baseline US and preoperative magnetic resonance. This technique, being dynamic and continuous, allows for a real-time direct view of the vascularization and flow distribution patterns of different types of neurosurgical lesions	CEUS adds valuable anatomic and biological information, such as vascularization, microcirculation, and tissue perfusion dynamics, which could possibly provide further insights into the pathology of brain tumors. It might help surgeons plan an approach to the lesion, highlight the lesion, distinguish between tumor and edematous brain tissue, and identify afferent and efferent vessels and hyperperfused areas, thereby possibly modifying the intraoperative surgical strategy
2014	Prada et al. (Italy) [29]	69	LGG22 HGG47	To perform the first characterization of cerebral glioma using CEUS and to possibly achieve intraoperative differentiation of different gliomas	CEUS is a fast, safe, dynamic, real-time, and economic imaging modality that might be helpful in differentiating malignant and benign gliomas during surgery and refining surgical strategies

HGG: high-grade glioma; LGG: low-grade glioma.

**Table 2:** Summary of the applications of three-dimensional ultrasound (3DUS) for glioma.

Year	First author and country	Patient no.	Grade	GTR (%)	Study endpoints	Results
2019	Bø et al. (Norway) [51]	74	LGG 74	30	To assess radiological and clinical results in consecutive patients with LGG treated with 3DUS-guided resection under general anesthesia	3DUS-guided LGG resections under general anesthesia are safe and they preserve HRQoL in most patients. Effectiveness in terms of EOR appears to be consistent with published studies using other advanced neurosurgical tools. Avoiding intraoperative vascular injury is a key factor for achieving good functional outcome
2018	Policicchio et al. (Italy) [52]	162	HGG 62 LGG 9 Others 91	54	To assess 3DUS visibility of different pathologies and IOUS applications during the course of surgery	IOUS was highly sensitive in detecting all types of pathology, was safe and precise in planning trajectories to intraparenchymal lesions (including minimally mini-invasive approaches), and was accurate in determining EOR in more than 80% of the cases. IOUS is a safe, versatile, and feasible tool that may be considered for routine intracranial surgery
2017	Šteňo et al. (Slovakia) [53]	28	LGG 28	86.79	To assess the effectiveness of 3DUS during awake resections of eloquent LGGs by comparing surgical results of two series of patients operated on using conventional neuronavigation and 3DUS	The extent of awake resections of eloquent LGG was greater with 3DUS guidance than with standard neuronavigation guidance; the use of 3DUS had no impact on the number of new permanent deficits
2017	Moiyadi and Shetty (India) [54]	22	HGG 17 LGG 5	78	To emphasize the convenience and feasibility of using navigable 3DUS with awake surgery for gliomas	Combining awake surgery with 3DUS is feasible and beneficial. It does not entail any additional surgical workflow modification or patient discomfort. This combined modality can be beneficial for eloquent region tumors
2016	Moiyadi and Shetty (India) [55]	111	HGG 75 LGG 12 Others 24	53	To evaluate the effectiveness of navigable 3DUS as a novel intraoperative imaging adjunct permitting quick real-time updates to facilitate tumor resection	The results of this study demonstrated that 3DUS can be effectively used as a stand-alone navigation modality during the resection of brain tumors. The ability to provide repeated, high-quality intraoperative updates is useful for guiding resection. Attention to image acquisition technique and experience can significantly increase the image quality, thereby improving the overall utility of this modality
2016	Arlt et al. (Germany) [9]	50	HGG 23 LGG 6 Others 21	62	To examine CEUS and 3DUS reconstructed ultrasound (3D-CEUS) during brain tumor surgery in terms of contrast agent uptake pre- and posttumor resection and imaging quality and compare them with postoperative MR imaging across different tumor entities	3D-CEUS is a reliable intraoperative imaging modality and could improve imaging quality. 90% of the high-grade gliomas showed high contrast uptake with improved imaging quality in more than 50%. GTR and incomplete resection of GBM were adequately highlighted by 3D-CEUS intraoperatively. CEUS can be a helpful imaging modality, especially for resection control in glioma surgery
2013	Moiyadi et al. (India) [56]	90	HGG 51 LGG 17 Others 22	67	To assess the practical utility of a navigable 3DUS system and its impact on intraoperative decisions during cerebral glioma surgery and analyze the EOR achieved in malignant gliomas	Navigable 3DUS is a versatile, useful, and reliable intraoperative imaging tool in resection of brain tumors, especially in resource-constrained settings where IOMR is not available. It has multiple functionalities that can be tailored to suit the procedure and the experience of the surgeon
2012	Sæther et al. (Norway) [44]	192	GBM 192	45 vs. 43 (with and without)	To examine if the introduction of 3DUS and neuronavigation may have had an impact on overall survival	Survival improved within the same period that IOUS and neuronavigation was introduced
2011	Rohde and Coenen (Germany) [57]	16	HGG 6 LGG 2 Others 8	/	To test if 3DUS likewise can be used for resection control	The number of investigated patients was too low to allow definite conclusions. However, the study results suggested that 3DUS is especially helpful for detecting overlooked brain tumor tissue
2008	Rygh et al. (Norway) [50]	19	HGG 19	76.9	To compare the ability of navigable 3DUS to distinguish tumor and normal brain tissue at the tumor border zone in subsequent phases of resection	The research showed that while ultrasound is highly accurate in delineating GBM before resection, it appears less accurate during and after resection. During resection, there seems to be some overestimation of the tumor, while small tumor remnants and infiltrated tissue in the cavity wall is underestimated after resection
2006	Lindner et al. (Germany) [58]	23	HGG 9 Others 14	77	To prove the concept of 3DUS in terms of technical effects and human impact. This includes measurement of fusion accuracy, extent of tumor resection, and the suitability for the detection and capture of intraoperative brain shift, as well as a protocol for operative handling as described by different neurosurgeons	The introduction of 3DUS substantially increased the value of neuronavigation, making several updates during surgery possible and minimizing problems related to brain shift
2005	Unsgaard et al. (Norway) [59]	28	HGG 15 LGG 7 Others 6	76.6	To compare interpretations of imaged biopsy sites with histopathology. The system also enabled concomitant comparison of navigated preoperative MR with histopathology	Reformatted images from 3DUS provides good delineation of metastases and solid glioma portions before starting the resection. Navigable 3DUS is at least as reliable as navigable 3D MR for delineating gliomas and metastases

GTR: gross total resection; HGG: high-grade glioma; LGG: low-grade glioma; MR: magnetic resonance.

**Table 3:** Summary of the applications of navigable ultrasound (NUS) for glioma.

Year	First author and country	Patient no.	Grade	GTR (%)	Study endpoint	Result
2019	Moiraghi et al. (Italy) [61]	31	HGG 31	61.2	To evaluate the impact of real-time conventional neuronavigation combining NUS and preoperative magnetic resonance imaging (MRI) on maximizing EOR in glioma surgery compared to standard conventional neuronavigation	The use of NUS-based real-time imaging modality promoted better EOR and neurological outcomes following the resection of noneloquent high-grade gliomas compared to standard conventional neuronavigation. NUS has proven to be useful in detecting RTV > 1 cm^3^
2019	Patil et al. (Indian) [67]	125	HGG 67 Others 58	—	To evaluate the relative utility and benefits of free-hand 2DUS and navigated 3DUS as ultrasound-guided biopsy techniques for supratentorial lesions	Despite the longer operative time and higher postoperative complication rates, NUS was beneficial for biopsies of deep-seated supratentorial lesions, while free-hand 2DUS remained valuable for superficial lesions
2016	Rueckriegel et al. (Germany) [68]	11	HGG 7 LGG 1 Others 3	27.27	To assess whether the combined use of navigated ultrasonography integrating FMRIB software library-based probabilistic fiber tracking into neuronavigation was technically feasible and achievable in the preoperative and intraoperative workflow	Integration of probabilistic fiber tracking and navigated ultrasonography into intraoperative neuronavigation facilitated anatomic orientation during glioma resection. Combination with NUS provided a three-dimensional estimation of intraoperative brain shift, thereby improving the reliability of neuronavigation
2014	Coburger et al. (Germany) [65]	15	GBM 15	75	To evaluate the use of NUS (linear array intraoperative ultrasound) for resection control in glioblastoma surgery	NUS (linear array intraoperative ultrasound) can be used as a safe and precise tool for intracranial image-guided resection control of GBM. NUS showed a significantly higher residual tumor detection rate compared to conventional imaging

RTV: residual tumor volume.

**Table 4:** Summary of the applications of ultrasound elastography (UE) for glioma.

Year	First author and country	Patient No.	Grade	Study endpoints	Results
2019	Cepeda et al. (Spain) [17]	36	HGG 4 LGG 16 Others 16	To determine the elastographic patterns of different brain tumor types and establish differences between their peritumoral regions	We objectively described the elastographic patterns of different types of brain tumors. We identified differences in both the tumors and peritumoral areas according to histologic types
2019	Prada et al. (Italy) [79]	64	HGG 38 LGG 7 Others 19	To describe the first large-scale implementation of strain elastography (SE) in oncological neurosurgery for lesions discrimination and characterization	SE allows clinicians to understand the mechanical properties of the brain and lesions during examination and permits better discrimination between different tissues compared to B-mode. Additionally, SE can differentiate between LGG and HGG
2016	Chauvet et al. (France) [78]	63	HGG 18 LGG 14 Others 31	To characterize elasticity of the normal brain parenchyma and brain tumors using shear-wave elastography (SWE)	Significant differences in elasticity were observed among the most common types of brain tumors. With intraoperative SWE, neurosurgeons may acquire innovative information to establish a diagnosis and guide resection

**Table 5:** Summary of the applications of functional ultrasound (FUS) for glioma.

Year	First author and country	Patient number	Grade	Study endpoints	Results
2017	Imbault et al. (France) [70]	33	LGG 33	We introduce a new, portable neuroimaging modality for the human brain based on FUS for deep functional cortical mapping	FUS identifies, maps, and differentiates regions of brain activation during task-evoked cortical responses within the depth of a sulcus in both awake and anesthetized patients

## Data Availability

The underlying data supporting the results of our study can be asked from the corresponding author.
